# Aggravation of Allergic Airway Inflammation by Cigarette Smoke in Mice Is CD44-Dependent

**DOI:** 10.1371/journal.pone.0151113

**Published:** 2016-03-21

**Authors:** Smitha Kumar, Ellen Lanckacker, Mieke Dentener, Ken Bracke, Sharen Provoost, Katrien De Grove, Guy Brusselle, Emiel Wouters, Tania Maes, Guy Joos

**Affiliations:** 1 Department of Respiratory Medicine, Laboratory for Translational Research in Obstructive Pulmonary Diseases, Ghent University Hospital, Ghent, Belgium; 2 Nutrition and Toxicology Research Institute Maastricht (NUTRIM), Department of Respiratory Medicine, University Hospital Maastricht, Maastricht, The Netherlands; Virginia Tech University, UNITED STATES

## Abstract

**Background:**

Although epidemiological studies reveal that cigarette smoke (CS) facilitates the development and exacerbation of allergic asthma, these studies offer limited information on the mechanisms involved. The transmembrane glycoprotein CD44 is involved in cell adhesion and acts as a receptor for hyaluronic acid and osteopontin. We aimed to investigate the role of CD44 in a murine model of CS-facilitated allergic airway inflammation.

**Methods:**

Wild type (WT) and CD44 knock-out (KO) mice were exposed simultaneously to house dust mite (HDM) extract and CS. Inflammatory cells, hyaluronic acid (HA) and osteopontin (OPN) levels were measured in bronchoalveolar lavage fluid (BALF). Proinflammatory mediators, goblet cell metaplasia and peribronchial eosinophilia were assessed in lung tissue. T-helper (Th) 1, Th2 and Th17 cytokine production was evaluated in mediastinal lymph node cultures.

**Results:**

In WT mice, combined HDM/CS exposure increased the number of inflammatory cells and the levels of HA and OPN in BALF and Th2 cytokine production in mediastinal lymph nodes compared to control groups exposed to phosphate buffered saline (PBS)/CS, HDM/Air or PBS/Air. Furthermore, HDM/CS exposure significantly increased goblet cell metaplasia, peribronchial eosinophilia and inflammatory mediators in the lung. CD44 KO mice exposed to HDM/CS had significantly fewer inflammatory cells in BALF, an attenuated Th2 cytokine production, as well as decreased goblet cells and peribronchial eosinophils compared to WT mice. In contrast, the levels of inflammatory mediators were similar or higher than in WT mice.

**Conclusion:**

We demonstrate for the first time that the aggravation of pulmonary inflammation upon combined exposure to allergen and an environmental pollutant is CD44-dependent. Data from this murine model of concomitant exposure to CS and HDM might be of importance for smoking allergic asthmatics.

## Introduction

Asthma, a chronic inflammatory disease of the airways, is often linked to allergen exposure. Sensitization to house dust mite (HDM) is a key predictive factor for asthma onset [1]. It is well known that exposure to mainstream or secondhand cigarette smoke (CS) not only increases the risk of asthma development, but also raises asthma-related morbidity and disease severity [2]. Despite several smoking prevention and cessation campaigns, the prevalence of cigarette smoking in asthmatics is as high as in the general population [3].

*In vivo* models that study asthma often only mimic allergen-induced lung inflammation. Animal models that combine allergen exposure with environmental pollutants such as CS better reflect the reality where (allergic) asthmatics are exposed to active and/or passive smoking in daily life. We described a murine model of CS-facilitated sensitization and aggravation of allergic airway inflammation to HDM [4] in which CS exposure increased many asthma features such as eosinophilia, Th2 cytokine production and goblet cell metaplasia. The molecular mechanisms leading to this CS-induced aggravation are however not yet unraveled.

CD44 is a transmembrane glycoprotein expressed by a wide variety of cells including structural and immune cells [5]. As a cell adhesion molecule, CD44 is involved in the migration and recruitment of leukocytes to sites of inflammation [6]. However, apart from its role in cell adhesion, CD44 can also function as a signaling receptor leading to pro-inflammatory chemokine expression by immune cells [7–9]. The expression of CD44 is shown to be increased in asthmatic epithelium [10] and increased CD44 levels have been demonstrated in serum of smokers [11]. However, Klagas and colleagues have demonstrated reduced CD44 expression on airway smooth muscle cells during asthma and COPD [12]. Using different antigens, Katoh and colleagues have elegantly demonstrated that CD44 is needed in the development of allergy [13–16]. In pulmonary fibrosis and hyperoxia induced lung injury models however, CD44 has a suppressive role on inflammation [17,18].

Hyaluronic acid (HA), a major ligand of CD44, is a high molecular weight (HMW) component of the extracellular matrix. Upon tissue damage or inflammation, HMW HA breaks down into low molecular weight (LMW) fragments which possess the ability to induce a number of inflammatory chemokines and cytokines [7] and activate inflammatory gene expression [19]. We have previously demonstrated enhanced LMW HA levels in lungs of CS exposed mice [20]. Airway secretions of asthmatics and COPD patients show higher HA levels [21,22]. Small HA fragments are implicated in the regulation of different murine models of allergic asthma [23,24].

Osteopontin (OPN), another major ligand of CD44, is also involved in the immune and inflammatory responses in asthma and cigarette smoking [25].

Although there are several reports of the importance of CD44, HA and OPN, either individually or as a receptor-ligand interaction, in allergic and non-allergic settings, molecular insights into the mechanisms by which environmental pollutants facilitate and aggravate the development of allergy are still lacking. We hypothesized that CD44 plays an important role in CS-facilitated allergic airway inflammation. To investigate this, we used a model of combined HDM and CS exposure to evaluate CS-aggravation of HDM-induced allergic inflammation in CD44 KO and WT mice. Our results indicate that the role of CD44 as an adhesion molecule is more important than its function as a signaling receptor in the recruitment of innate immune cells and the development of a Th2 response in CS-aggravated allergic airway inflammation.

## Materials and Methods

### Mice

Male C57BL/6 WT mice were purchased from the Jackson Laboratories. CD44 KO breeding pairs were purchased from the Jackson Laboratories and bred at the animal breeding facility of the Faculty of Medicine, Ghent University. Animals aged 8–10 weeks were included in the experiment. All *in vivo* manipulations were approved by the Local Ethics Committee for animal experimentation of the Faculty of Medicine, Ghent University (ECD 14/07). The experiments were carried out in accordance with the approved guidelines of the Faculty of Medicine and Health Sciences (Ghent, Belgium).

### Cigarette Smoke (CS) exposure and House Dust Mite (HDM) administration

**Fig 1:**
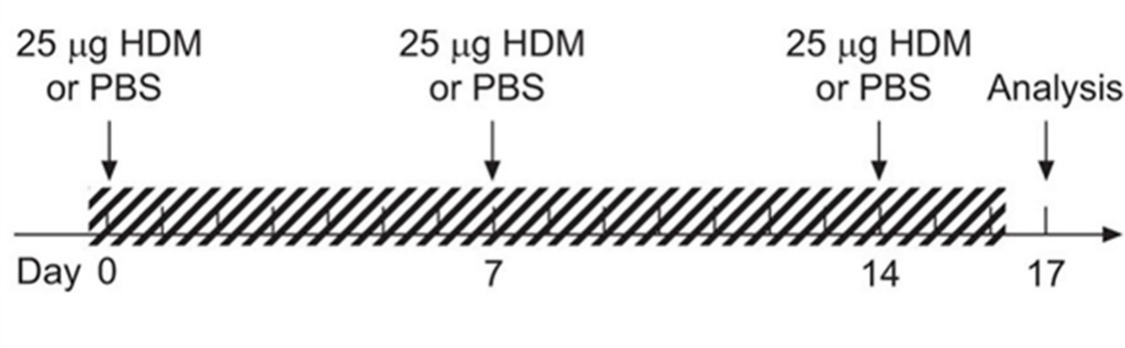
Exposure protocol of CS and HDM or PBS (adapted from [4]). The shaded region represents CS or air exposure.

Mice (n = 8-10/group) were subjected whole body to CS for 3 consecutive weeks as described previously [4]. Briefly, mice were exposed to tobacco smoke of 5 cigarettes (Reference Cigarette 3R4F without filter) 3 times a day with 30-minute smoke-free intervals 5 days per week for 3 weeks. Control mice were exposed to air. Once a week, 30 minutes after the last CS or air exposure, 25 μg HDM extract (Greer Laboratories) or PBS was administered intranasally in isoflurane anesthetized mice (Fig 1).

### Bronchoalveolar Lavage Fluid (BALF) and differential cell counts

Twenty-four hours after the last CS exposure and 72 hours after the last HDM administration, mice were euthanized with an overdose of pentobarbital (Sanofi-Ceva). BALF recovery and differential cell counts were performed as described previously [20]. Briefly, a tracheal cannula was inserted, and BALF was recuperated by instillation of 3 × 300 μl HBSS without Ca^2+^ or Mg^2+^ (BioWhittaker, Lonza, Basel, Switzerland) supplemented with 1% BSA (for cytokine and chemokine measurements; Sigma-Aldrich, St. Louis, MO) and 6 × 500 μl HBSS without Ca^2+^ or Mg^2+^ supplemented with 0.6 mM sodium EDTA (Sigma-Aldrich). For each mouse, cells from the different lavage fractions were pooled and total cell counts were determined using a Bürcker chamber. Remaining cells were used for flow cytometric analysis.

### Flow cytometry

Cells were labeled with fluorochrome conjugated monoclonal antibodies against the following cell markers: CD11c, MHC class II (MHC-II), CD11b, Ly6C, Ly6G, Siglec-F, CD3, CD4, CD8 and CD44 (BD Pharmigen). 7-Aminoactinomycin D (BD Pharmigen) was used for dead cell exclusion. Cells were labeled with various combinations of mAbs. Dendritic cells were characterized as CD11c^+^ low autofluorescent, MHCII^+^, CD11b^+^ cells [26,27]. Neutrophils were characterized as CD11c^−^CD11b^+^Ly6C^+^Ly6G^+^ cells. Eosinophils were characterised as CD11c^−^CD11b^+^Siglec F^+^ cells. T-cells are identified as CD3^+^ cells with low side scatter. They are further gated as either CD4^high^ or CD8^high^ [4]. Data acquisition was performed on a FACSCalibur flow cytometer running CellQuest software. FlowJo software (Tree Star) was used for data analysis.

### Mediastinal lymph node culture

Paratracheal and parathymic lymph nodes were collected in sterile tubes containing tissue culture medium [4] and digested to obtain a single cell suspension. Briefly, minced lymph nodes were incubated with 1 mg/ml collagenase (Worthington Biochemical, Lakewood, NY) and 20 μg/ml DNAse I (Boehringer Mannheim, Brussels, Belgium) for 45 min at 37°C and 5% CO_2_. Red blood cells were lysed using ammonium chloride buffer. Finally, cell suspensions were filtered through a 50 μm nylon mesh to remove undigested organ fragments. Cells were then transferred in triplicate to round-bottom, 96-well plates (Becton Dickinson) with or without 15μg HDM extract/ml culture medium at a density of 2 x 10^5^ cells per well and incubated in a humidified 37°C incubator with 5% CO_2_. After 5 days, supernatant was harvested and frozen for cytokine measurements.

### Measurement of airway hyperresponsiveness

Lung function measurements were made as described previously [4]. Briefly, airway resistance was measured invasively in anaesthetized tracheostomized mice using the Flexivent system (SCIREQ). Neuromuscular blockade was induced using intravenously injected pancuronium bromide (1 mg/kg). Mice were challenged with increasing doses of carbachol. The resistance (R) of the airways, lung and chest wall was measured. A dose-response curve was generated for each mouse.

### Cytokine measurements

Lung tissue homogenate was prepared as described previously [28]. ELISAs for IFN-γ, IL-4, IL-5, IL-13 and IL-17 in the mLN culture supernatant, IL-1 β and IL-33 in lung homogenate and HA and OPN in BALF were performed according to the manufacturer’s guidelines (all R&D Systems except HA, Tebu-bio).

### Histology

The left lung was intratracheally infused with 4% paraformaldehyde for tissue fixation. Transversal sections of 3μm were stained with Congo Red to stain eosinophils. Periodic acid-Schiff (PAS) stain was used to identify goblet cells. Goblet cells in airway epithelium and peribronchial infiltration of eosinophils were quantified using a Zeiss KS400 image analyzer platform (KS400, Zeiss) as described previously [29]. Lung tissue was stained with haematoxylin-eosin for general inflammation pathology scoring, which was performed by two independent observers on blinded lung tissue sections. A score was assigned between 0 and 3, based on the average peribronchial inflammatory cells for each mouse (score 0: 0–5 inflammatory cells; 1: 5–20 inflammatory cells, 2: 20–50 inflammatory cells; 3: >50 inflammatory cells).

### RNA isolation and qRT-PCR

Total lung RNA was extracted with the RNeasy Mini Kit (Qiagen). cDNA was prepared using Transcriptor First Strand cDNA synthesis kit (Roche Diagnostics). Expression of target genes CXCL1 and CCL11 and reference genes HPRT-1, GAPDH and TFRC were measured using Taqman Gene Expression assays (Applied Biosystems). Real-time PCR was performed in duplicate using 10 ng cDNA with a LightCycler96 detection system. Data was processed using the standard curve method. Expression of target genes was normalized based on the three reference genes [30].

### Statistical analysis

Reported values are expressed as mean ± SEM. Statistical analysis was performed with IBM SPSS Statistics 22. 0 using nonparametric tests (Kruskall-Wallis and Mann-Whitney U). P values under 0.05 were considered significant.

## Results

### Cigarette smoke facilitates house dust mite-induced airway inflammation in C57BL/6 mice and increases Hyaluronic Acid (HA) levels in BALF

**Fig 2:**
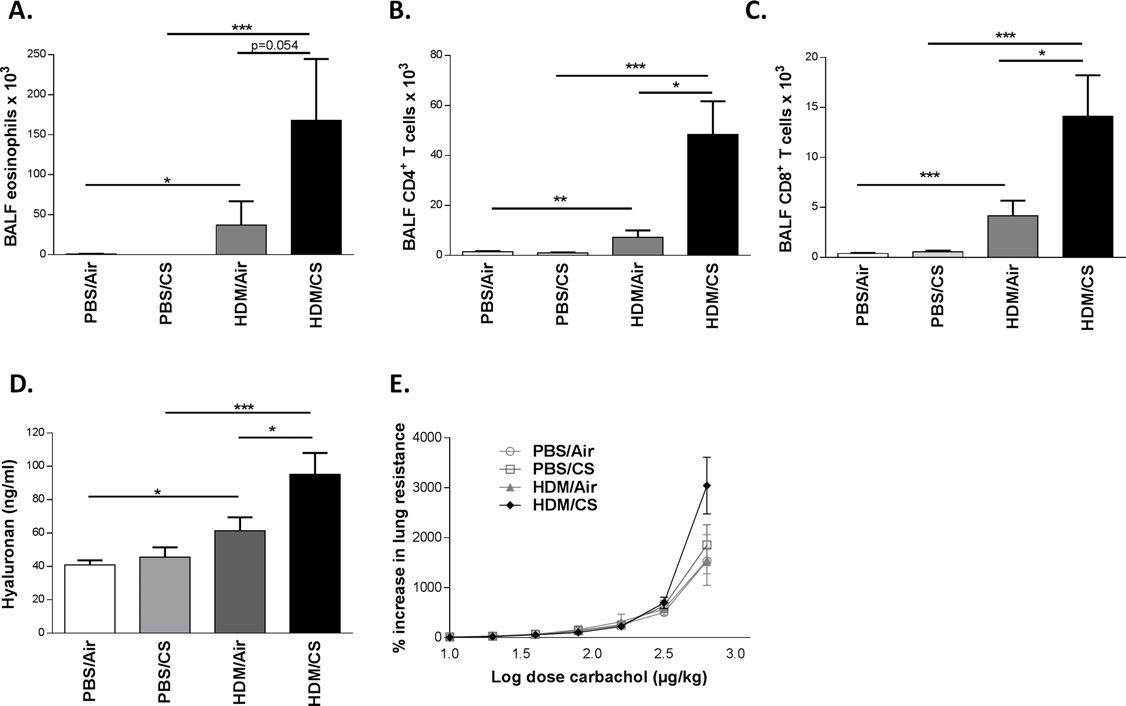
Effect of combined HDM/CS exposure in C57BL/6 mice. **A-C:** Total cell numbers of eosinophils, CD4^+^ and CD8^+^ T lymphocytes respectively in BALF of WT C57BL/6 mice exposed to PBS or HDM combined with CS or air for 3 weeks; n:8–10 mice/group). **D:** HA levels in BALF of WT C57BL/6 mice exposed to PBS or HDM combined with CS or air for 3 weeks (graph representative for 3 independent experiments). **E**: Dose-response curve of airway hyperresponsiveness to carbachol in WT C57BL/6 mice exposed to PBS or HDM combined with CS or air for 3 weeks. *p<0.05, **p<0.01, ***p<0.005; n = 8–10 mice/group.

**Fig 3:**
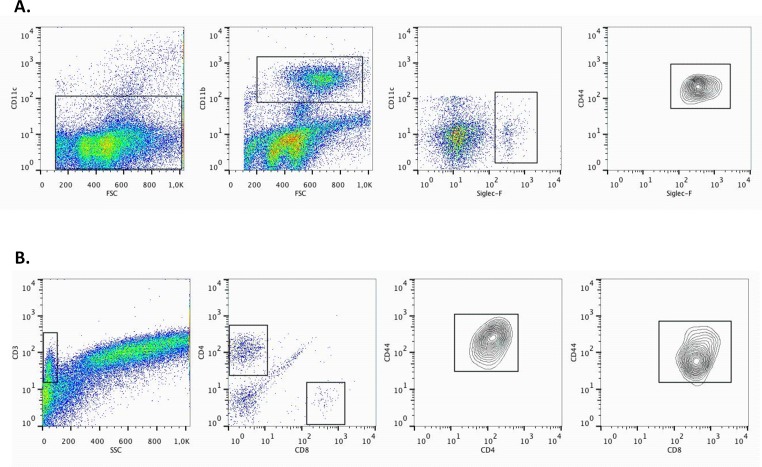
CD44 positivity of eosinophils, CD4^+^ and CD8^+^ T lymphocytes of WT C57BL/6 mice exposed to HDM and CS for 3 weeks. **A:** Gating strategy for identifying eosinophils. CD11c^low^, CD11b ^high^, Siglec-F^high^ cells are identified as eosinophils. They are further characterized as being CD44^high^. **B:** Gating strategy for identifying CD4^+^ and CD8^+^ T lymphocytes. T cells are identified as CD3^positive^ cells with low side scatter. They are further gated as either CD4^high^ or CD8^high^. Both CD4^+^ and CD8^+^ T lymphocytes are CD44^positive^.

We previously reported a murine model in Balb/c mice in which cigarette smoke (CS) facilitates house dust mite (HDM)-induced allergic airway inflammation whereas sole HDM exposure induces only mild inflammation [4]. In order to perform mechanistic studies, we first investigated whether this model was transposable to C57BL/6 mice, which are known to be less Th2-prone [31]. As in the Balb/c mice, CS aggravated the HDM-induced inflammation characterized by increased number of eosinophils, and CD4^+^ and CD8^+^ lymphocytes in brochoalveolar lavage fluid (BALF) (Fig 2A–2C). All the above mentioned immune cells were CD44 positive (Fig 3).

Since elevated hyaluronic acid has been described in patients with asthma, we measured HA levels in the combined HDM/CS model. In three independent experiments, simultaneous exposure to CS and HDM increased the levels of soluble HA in BALF compared to HDM/Air or PBS/CS exposure (Fig 2D and data not shown).

Airway hyperresponsiveness is a characteristic feature of asthma. Fig 2E shows the dose–response curve of airway reactivity in C57BL/6 mice. Although C57BL/6 mice exposed to both HDM and CS show a trend towards increased airway hyperresponsiveness, there were no significant differences between the four groups, in contrast to our previous data in Balb/c mice [4].

### Both CS-induced inflammation and CS-aggravated allergic inflammation are attenuated in CD44 KO mice

**Fig 4:**
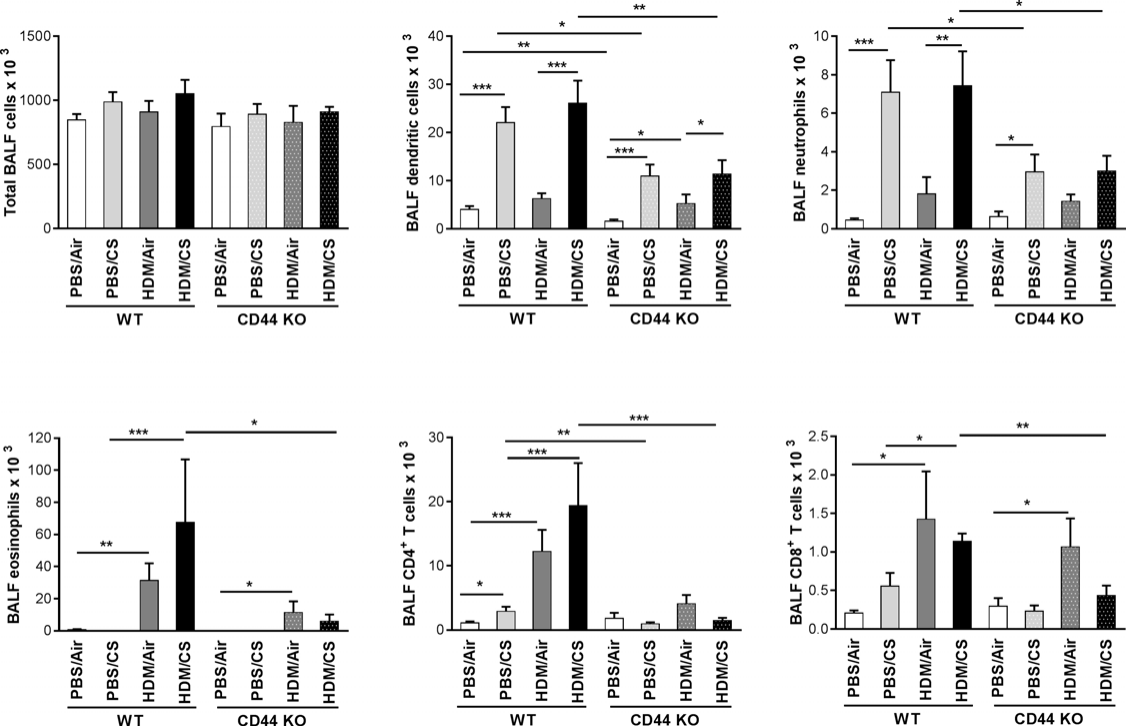
Effect of CD44 on inflammation upon combined HDM/CS exposure. Total cell numbers of dendritic cells (low autofluorescent CD11c^+^, CD11b^+^, MHCII^+^), neutrophils (CD11c^−^CD11b^+^Ly6C^+^Ly6G^+^), eosinophils (CD11c^−^CD11b^+^Siglec F^+^), CD4^+^ and CD8^+^ T lymphocytes in BALF of WT and CD44 KO mice exposed to PBS or HDM combined with CS or air for 3 weeks. *p<0.05, **p<0.01, ***p<0.005; n = 10 mice/group.

To investigate the role of CD44, a major HA receptor, in the CS-aggravated allergic response, we exposed WT and CD44 KO mice to the combined HDM/CS exposure protocol and focused on the inflammatory response. Although total cell numbers in BALF were similar, combined HDM/CS exposure significantly attenuated the number of dendritic cells, neutrophils, eosinophils and CD4^+^ and CD8^+^ lymphocytes in CD44 KO mice compared to WT mice. Notably, the number of these cells did not differ significantly between WT and CD44 KO mice exposed to HDM alone. Upon PBS/CS exposure, the number of dendritic cells, neutrophils and CD4^+^ lymphocytes were also significantly reduced in CD44 KO mice compared to PBS/CS-exposed WT animals (Fig 4). Exposure to CS increased the MHCII expression within the CD11c^+^, low autofluorescent, CD11b^+^ dendritic cells in both WT C57/Bl6 and CD44 KO mice, without any differences between the two strains (S1 Fig).

### Attenuated Th2, but not Th1 and Th17 cytokine production in CD44 KO mice simultaneously exposed to HDM/CS

**Fig 5:**
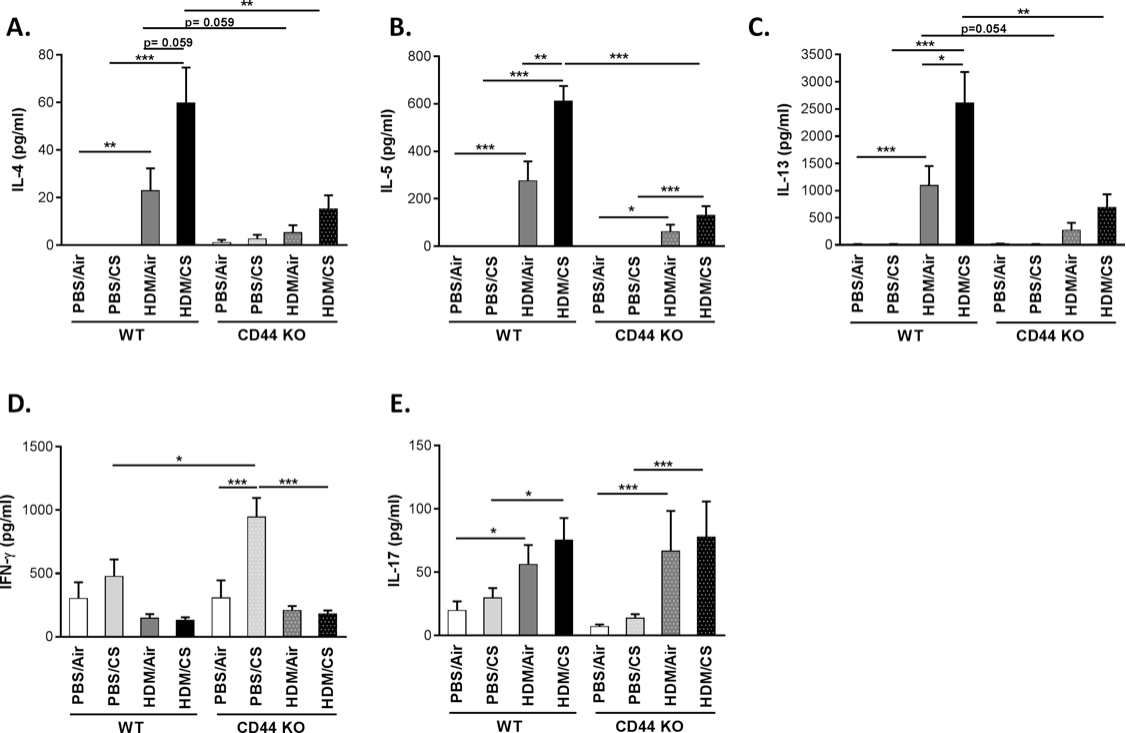
Effect of CD44 on lymph node cytokine production upon combined HDM/CS exposure and HDM restimulation. **A-E:** Cytokine levels in supernatant of mediastinal lymph node cell cultures from WT and CD44 KO mice that were exposed for 3 weeks to PBS/air, PBS/CS, HDM/air or HDM/CS, upon restimulation with HDM. **A-C**: Th2 cytokines (IL-4, IL-5 and IL-13). **D:** Th1 cytokine (IFN-γ). **E:** Th17 cytokine (IL-17). *p<0.05, **p<0.01, ***p<0.005; n = 8–10 mice/group.

Allergic asthma is associated with increased levels of Th2 cytokines. To evaluate whether Th2 cells are present in the lymph nodes in our model after 3-weeks exposure to either PBS/air, PBS/CS, HDM/air or HMD/CS, we isolated mediastinal lymph node cells and restimulated them *in vitro* with HDM to evaluate Th2 cytokine (IL-4, IL-5 and IL-13) production [32] In the combined HDM/CS exposure groups, the CS-aggravated release of Th2 cytokines was significantly diminished in the CD44 KO compared to WT mice. In CD44 KO mice exposed to HDM/Air, IL-4, IL-5 and IL-13 tended to be reduced compared to WT mice (Fig 5A–5C).

In lymph nodes cultures without HDM restimulation, IL-4 and IL-5 levels were undetectable (data not shown) whereas low levels of IL-13 (20x lower compared to HDM-stimulated lymph node cultures) were only detectable in cultures from HDM/air or HDM/CS exposed mice (both in WT and CD44 KO) (S2 Fig).

CD44 KO mice exposed to PBS/CS mounted a significantly higher Th1 response (IFN-γ) compared to PBS/CS exposed WT mice. Interestingly, CD44 KO mice simultaneously exposed to HDM and CS failed to show this response. There were no differences between the four groups in the WT animals. IFN-γ levels were highest in the PBS/CS exposed group in the CD44 KO mice (Fig 5D).

The Th 17 response was similar in WT and CD44 KO mice. Simultaneous exposure to HDM and CS significantly increased IL-17 levels compared to PBS/CS in both WT and CD44 KO mice. However, this did not differ significantly from HDM/Air exposure (Fig 5E).

In lymph nodes cultures without HDM restimulation, there were no significant differences in IFN-γ and IL-17 production between the 8 experimental groups (S2 Fig).

### CS-aggravated goblet cell metaplasia and peribronchial eosinophil infiltration is attenuated in CD44 KO mice

**Fig 6:**
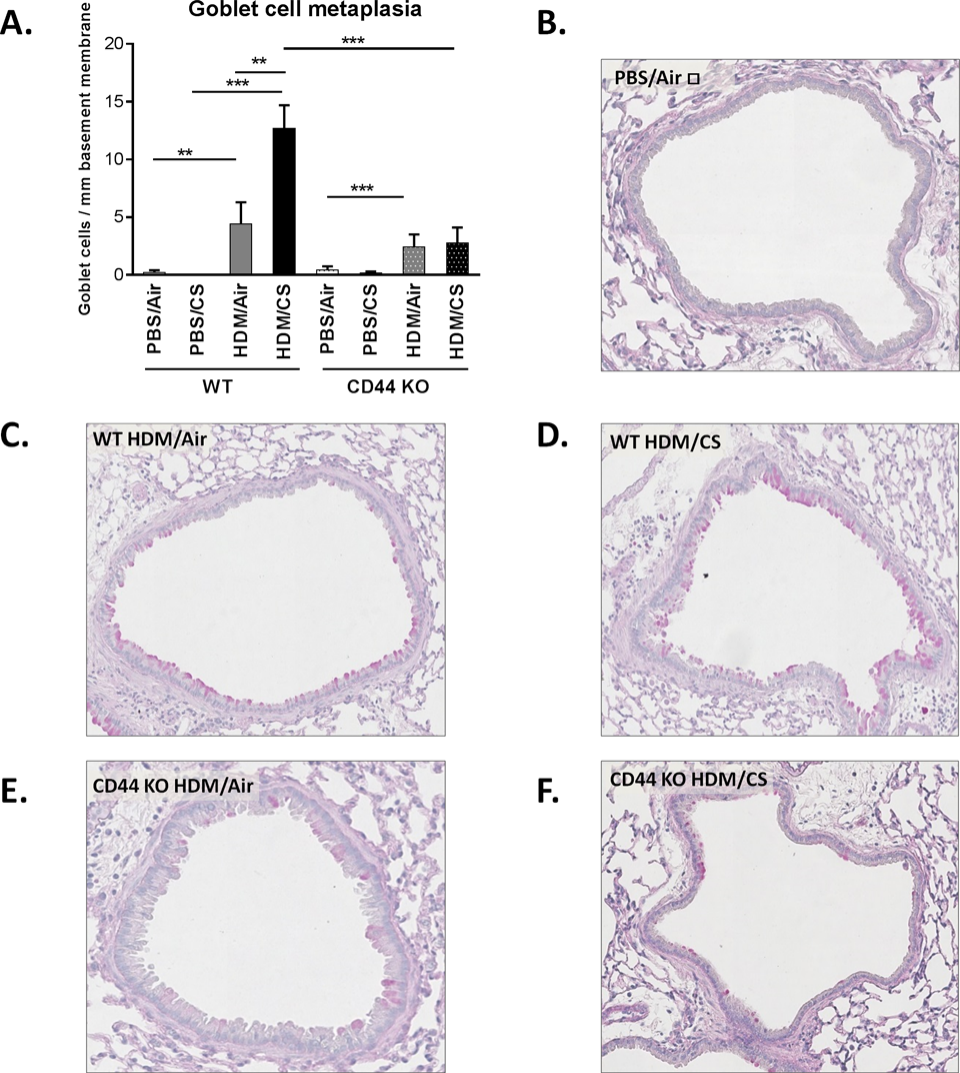
Effect of CD44 on goblet cell formation upon combined HDM/CS exposure. Quantification of mucus producing cells in WT and CD44 KO mice (**A**). Periodic acid-Schiff (PAS) stain was used to identify goblet cells in the airway mucosa (**B-F**).ⱡ: Figure representative of PBS/Air and PBS/CS of WT and CD44 KO mice. *p<0.05, **p<0.01, ***p<0.005; n = 8–10 mice/group.

**Fig 7:**
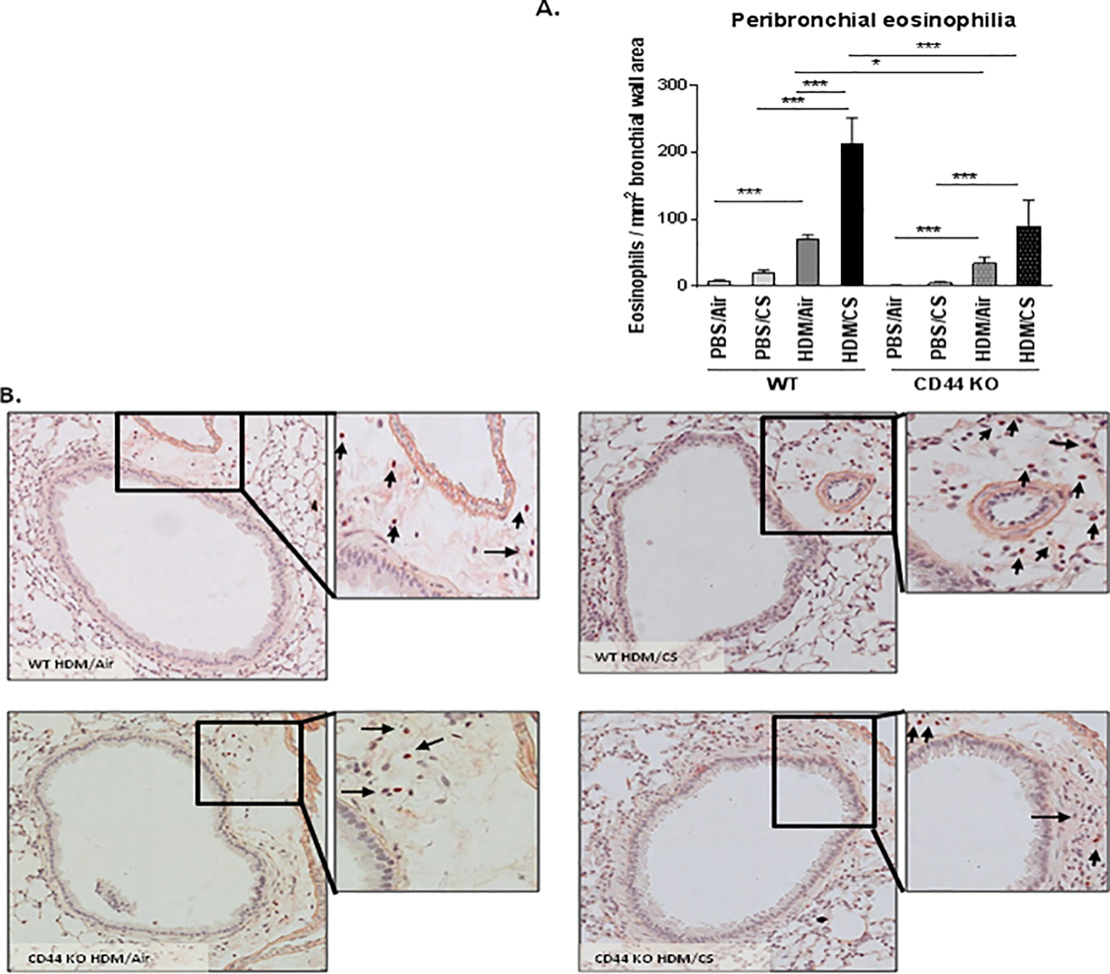
Effect of CD44 on airway wall eosinophilia upon combined HDM/CS exposure. Quantification of eosinophils in airway wall in WT and CD44 KO mice (**A**). Congo Red staining was used to identify eosinophils in bronchial wall (**B**). *p<0.05, **p<0.01, ***p<0.005; n = 8–10 mice/group.

We also evaluated two hallmarks of allergic airway inflammation, namely increased number of mucus producing cells and eosinophil infiltration in the bronchial walls. In CD44 KO mice, the HDM/CS exposure group showed significantly decreased goblet cell metaplasia (Fig 6) and peribronchial eosinophilic infiltrate (Fig 7) as compared to WT animals. Upon HDM/Air exposure, the modest increase in goblet cell metaplasia did not differ between WT and CD44 KO mice, whereas the peribronchial eosinophilia was attenuated in CD44 KO mice. Semi-quantitative pathology grading on haematoxylin-eosin stained lung tissue demonstrated a decreased pathology score in CD44 KO mice compared WT mice upon HDM/CS exposure, yet no CS-aggravating effect was detectable with this scoring method (S3 Fig).

### Hyaluronic acid and osteopontin levels in BALF of CD44 KO mice

**Fig 8:**
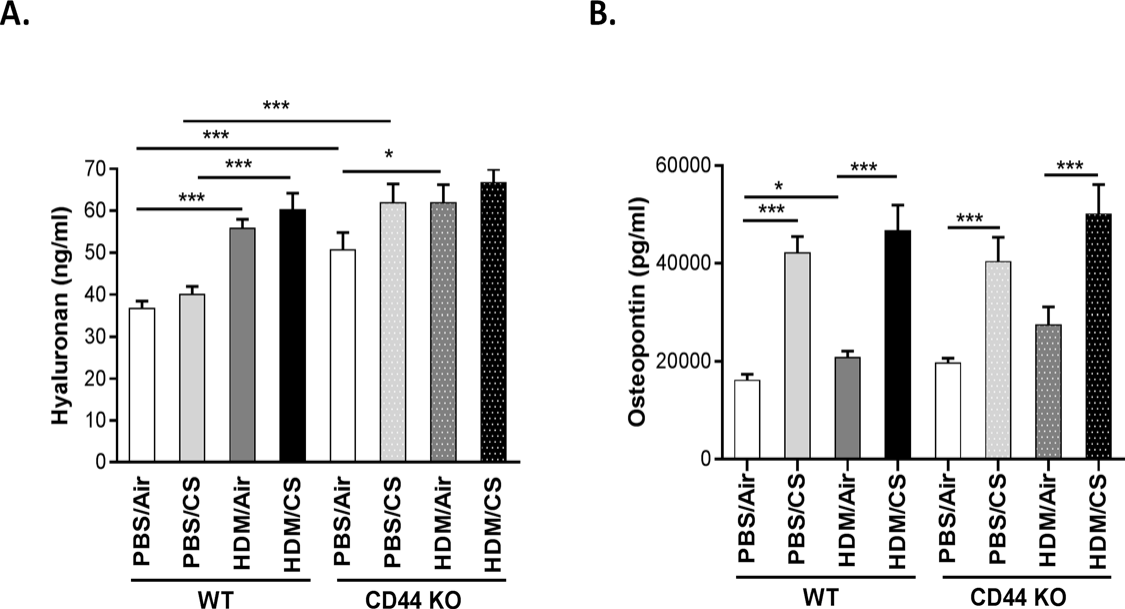
Effect of CD44 on hyaluronic acid and osteopontin levels upon combined HSM/CS exposure. HA (A) and OPN (B) levels in BALF of WT and CD44 KO mice that were exposed for 3 weeks to PBS/air, PBS/CS, HDM/air or HDM/CS. *p<0.05, **p<0.01, ***p<0.005; n = 8–10 mice/group.

In WT mice, the HA levels in BALF were highest in the HDM/CS group (Fig 8A). Basal HA levels were higher in the BALF of CD44 KO mice. Compared to the PBS/Air group, all other groups had elevated HA levels in the CD44 KO mice although there were no significant differences in HA levels between PBS/CS, HDM/Air or HDM/CS groups.

The expression pattern of OPN was similar in WT and CD44 KO mice. Simultaneous exposure to HDM and CS significantly increased OPN levels compared to HDM/Air in both WT and CD44 KO mice. However, this did not differ significantly from PBS/CS exposure (Fig 8B).

### Pro-inflammatory mediator expression in lungs of WT and CD44 KO mice

**Fig 9:**
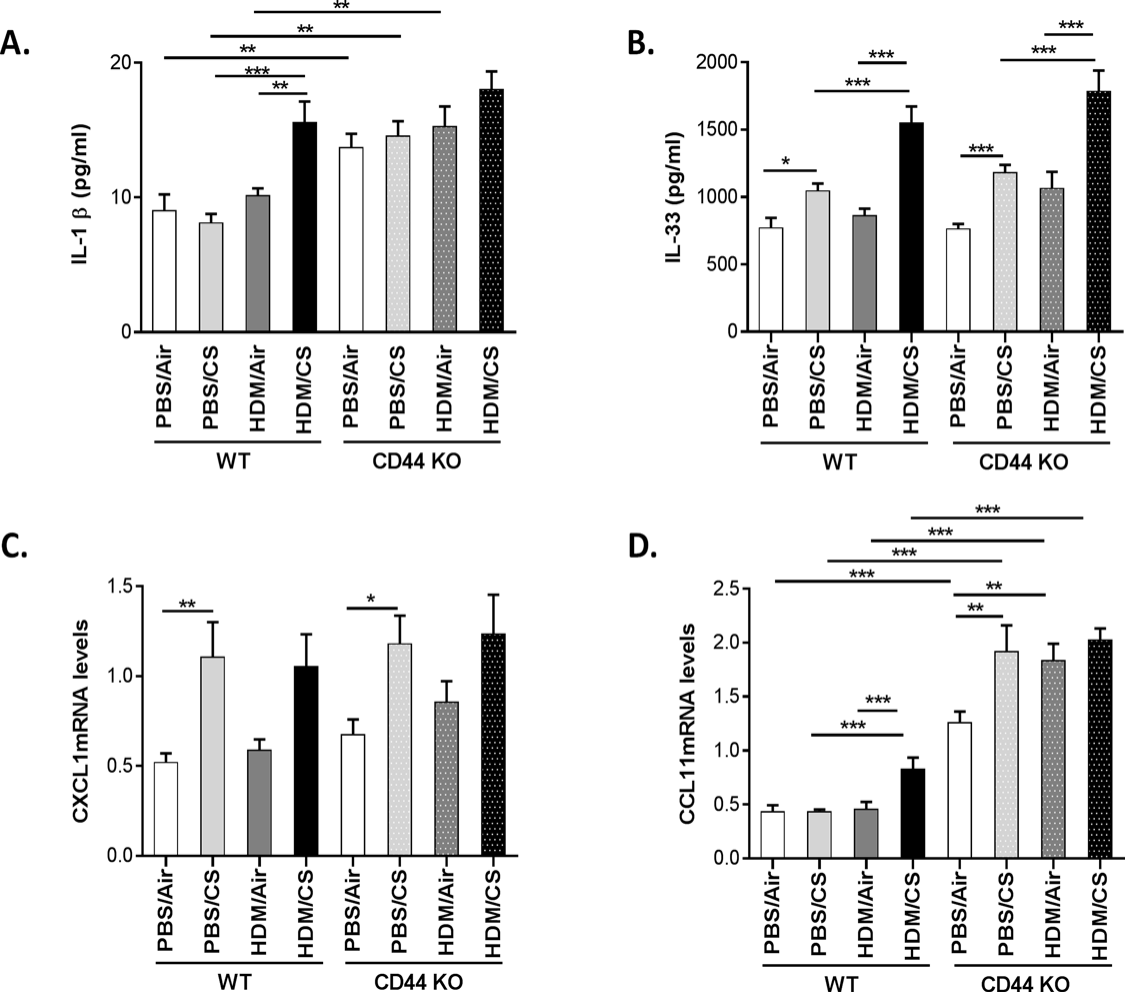
Effect of CD44 on proinflammatory mediator production upon combined HDM/CS exposure. Proinflammatory mediator levels in lungs of WT and CD44 KO mice. IL-1β (A) and IL-33 (B) protein levels and CXCL1 (C) and CCL11 (D) mRNA expressions. *p<0.05, **p<0.01, ***p<0.005; n = 10 mice/group.

WT mice simultaneously exposed to HDM and CS showed significantly enhanced protein levels of IL-1 β and IL-33 as compared to the control groups (Fig 9A and 9B). CXCL1 mRNA expression was increased in WT mice in both PBS/CS and HDM/CS groups, whereas CCL11 mRNA expression was only significantly increased in the combined HDM/CS exposure group (Fig 9C and 9D). In CD44 KO mice, the expression patterns of IL-33 and CXCL1 were similar to WT mice (Fig 9). Notably, the baseline levels of IL-1 β and CCL11 were higher in CD44 KO compared to WT. The exposure to CS, HDM or the combination significantly increased CCL11 expression in CD44 KO mice (Fig 9).

## Discussion

Exposure to environmental pollutants like cigarette smoke is considered to be an important risk factor for the development and aggravation of asthma. However, the molecular mechanisms leading to this CS-induced aggravation remain poorly understood. In a murine model of combined exposure to allergens and cigarette smoke—mimicking smoking allergic asthmatics -we observed increased hyaluronic acid (HA) levels in the combined HDM/CS exposure group as compared to HDM or CS alone, suggesting a putative role for this molecule in the inflammatory process. Compelling evidence suggests that HA works through its receptor CD44 to regulate allergic airway inflammation. Although there are reports of the importance of HA and/or CD44 in allergic inflammation initiated by various allergens like cockroach antigen and ovalbumin [23,24], all studies were performed in the absence of any environmental pollutant. Since, depending on the model, CD44 can have a proinflammatory or immunosuppressive role [13,17,18,33,34] we investigated the role of CD44 in a murine model of HDM-induced allergic airway inflammation aggravated by CS exposure. To our knowledge, our study is the first to report an attenuation of the aggravating effect of CS on allergic inflammation to HDM in CD44-deficient mice.

We have previously reported that CS aggravates the allergic asthma phenotype in Balb/c mice [4,35]. Earlier work from our lab has demonstrated the importance of genetic background on allergic phenotype [29]. C57BL/6 mice are known to be less Th2-prone and to develop less airway hyperresponsiveness compared to the Balb/c strain [31]. In order to perform mechanistic studies with knock-out animals, most of which have a C57BL/6 background, we first explored whether the Balb/c mouse model was transposable to the C57BL/6 strain. As in the Balb/c mice, CS aggravated the HDM-induced allergic inflammation in the C57BL/6 strain, although the inflammation was less pronounced than in Balb/c mice and there was some inter-experimental variation in the responses. C57BL/6 mice exposed simultaneously to HDM/CS showed a trend towards increased hyperresponsiveness, but there were no significant differences between the four groups. Therefore, we did not investigate lung function upon HDM/CS exposure in CD44 KO mice.

CD44 deficiency has been implicated in decreased Th2-mediated airway inflammation and airway hyperresponsiveness in antigen-sensitized mice [16]. To investigate whether CD44 is important in the context of an aggravated inflammatory response due to environmental pollutants, we examined CS-induced allergic inflammation in WT and CD44 KO mice. We demonstrate that CD44 KO mice are protected against worsening of allergic pulmonary inflammation induced by the combination of HDM and CS. CD44 KO mice showed significantly reduced numbers of inflammatory leukocytes in BALF upon combined HDM/CS exposure. Inflammatory cell influx into lungs is an important process during pulmonary inflammation. Several studies suggest an important role for CD44 during infectious and sterile lung injury. CD44 KO mice were protected from during ozone-induced airway hyperresponsiveness [33]. On the other hand, in a model of pulmonary fibrosis, CD44 KO mice demonstrated enhanced and unresolved inflammation following intratracheal administration of bleomycin [18]. This difference in the effects of CD44 on pulmonary inflammation in different models appears to be dependent on the administered stimulus and the severity of tissue damage. Our HDM/CS model in C57/Bl6 has a modest inflammation, without increases in typical markers of injury such as albumin and lactate dehydrogenase in BALF (data not shown).

Th2 cytokines play an essential role in the pathogenesis of allergic airway inflammation. While IL-4 and IL-5 promote isotype switching of B cells to IgE producing cells and the infiltration of target tissues by eosinophils, IL-13 induces metaplasia of epithelial cells into goblet cells which is responsible for mucus hypersecretion in allergic asthma. In the combined HDM/CS exposure groups, the CS-aggravated release of Th2 cytokines was significantly reduced in the CD44 KO lymph node cultures compared to WT, although a limited response remained present. In line with the Th2 cytokine levels, CD44 KO mice exposed to combined HDM/CS presented significantly decreased goblet cell metaplasia and peribronchial eosinophilic infiltrate. We also investigated Th1 and Th17 responses in lymph node cultures of WT and CD44 KO mice, by measuring IFN-γ and IL-17, respectively. WT mice showed no significant differences in IFN-γ production upon different exposures, although the levels tended to be highest in the PBS/CS group. Interestingly, PBS/CS exposure in CD44 KO mice yielded in a significantly higher Th1 response (IFN-γ) compared to WT mice and CD44 KO mice simultaneously exposed to HDM/CS failed to show this response. The Th2 hypothesis for asthma describes that asthma is caused by an increased Th2 response in combination with a decreased Th1 response [36]. The lower levels of IFN-γ in HDM and HDM/CS exposed animals indicate indeed the shift to a Th2 response.The decrease in CD4^+^ and CD8^+^ T cells in BAL and Th2 cytokines in the lymph nodes in CD44 KO mice could result from a decreased dendritic cell migration and/or maturation. Indeed, CD44 blocking experiments have demonstrated that CD44 modulates dendritic cell maturation/migration and the induction of T-cell responses [37,38]. On the other hand, CD44 expression on Ag-specific T-cells also determines its interaction with dendritic cells and resulting T-cell expansion [39]. In current work, we did not observe differences in the levels of MHCII expression between wild type and CD44 KO animals. Although MHCII is marker associated with dendritic cell maturation/activation [40] future research investigating specific dendritic cell maturation markers (e.g. CD86) and migration in CD44 KO mice may shed more light on the resulting decrease in the T helper cytokine response in our model. Of note, in Balb/c we have previously demonstrated increased dendritic cell maturation and migration to regional lymph nodes in the combined HDM/CS model [4].

Recently, the role of Th17 response in asthma has been a subject of intense investigation. IL-17 has been found in blood and BAL fluid from asthmatic patients and levels of IL-17 in the airways correlate with asthma severity [41,42]. IL-17-deficient mice show defective allergen specific T cell activation and reduced airway hyperresponsiveness [43]. In our study, simultaneous exposure to HDM and CS significantly increased IL-17 levels compared to PBS/CS in both WT and CD44 KO mice. However, this did not differ significantly from HDM/Air exposure. The Th17 immunity seems to be CD44 independent since both WT and CD44 KO mice demonstrate a similar Th17 response. Our data confirm the findings of Schnyder-Candrian and colleagues who have already shown an enhanced IL-17 production by cells from mediastinal lymph nodes in an asthma model [44].

The effects of CD44 deficiency were less pronounced upon sole HDM exposure. IL-4, IL-5 and IL-13 tended to be reduced in CD44 KO mice exposed to HDM/Air. Although there was a significant decrease in lung eosinophil number in CD44 KO mice, there were no significant differences between the inflammatory cells in BALF and number of goblet cells. These data are not in full accordance to previously described allergic models where decreased inflammation was reported upon CD44 deficiency [13,14], but variations in the inflammatory responses in CD44 KO mice can be attributed to the dissimilarities in the used allergen, the dose and experimental protocol. In our experimental model, only a modest allergic response to HDM is observed in the absence of the aggravating factor CS.

Notably, the effects of sole CS exposure (increased number of dendritic cells, neutrophils and CD4^+^ lymphocytes) were also significantly attenuated in CD44 KO mice. It may be of interest to examine the involvement of CD44 in the pathogenesis of COPD, a disease caused by chronic CS exposure.

Hyaluronic acid has been described as an inflammatory mediator in patients with asthma and its levels have been correlated with disease severity [21,45]. Increased HA levels in BALF during airway inflammation have also been reported in mouse models [33,46]. We measured whether HA levels were modulated in the combined HDM/CS model in C57BL/6 mice. Although we observed some experimental variation, simultaneous exposure to CS and HDM increased the levels of soluble HA in BALF compared to HDM/Air or PBS/CS exposure in 3 independent experiments. The increased ligand (HA) levels associated with increased inflammation in WT mice, combined with an attenuated inflammatory response in the absence of the receptor (CD44) suggest that the HA-CD44 interaction could be implicated in the aggravated allergic response to HDM upon exposure to CS. However, due to limited amounts of lung tissue and BAL, we could not demonstrate that the increased soluble HA levels are proinflammatory short HA fragments (data not shown). In addition, we did not provide direct mechanistic evidence that the HA-CD44 interaction drives the aggravated allergic response to HDM upon exposure to CS. Further research using HA-inhibitors, as has been done in an ozone-induced model of airway hyperresponsiveness [33] could provide more insights into this matter. Notably, CD44 KO mice had elevated baseline levels of soluble HA which further increased upon CS or HDM exposure. Such increased concentrations of HA in BALF of CD44 KO mice has previously been attributed to reduced clearance of HA fragments in the absence of the HA receptor CD44 [47,48].

Osteopontin, another major ligand of CD44, plays an important role in the pathogenesis of Th2 skewing witnessed in allergic asthma [49]. To determine whether OPN is involved in CS- aggravated allergic inflammation, we measured OPN concentration in BALF. Although the OPN levels were significantly increased in the HDM/CS exposure group, this was mainly a CS effect as there was no significant difference with the sole CS group. Notably, OPN expression was also CD44 independent. However, this does not exclude the role of OPN in the aggravation of allergic pulmonary inflammation upon CS exposure. Future experiments using OPN knockout mice could elucidate this further.

Chemokines and cytokines play an important role in the recruitment of inflammatory cells upon tissue damage. To evaluate whether an impaired chemokine production lies at the basis of the decreased inflammatory response in CD44 KO mice, we measured the levels of CXCL1, a potent recruiter of neutrophils, CCL11, an eosinophil chemoattractant, IL-33, an epithelial cytokine and IL-1 β in lung. Remarkably, this proinflammatory mediator production was CD44 independent. Despite higher cytokine and chemokine levels, the inflammatory cell recruitment in the CD44 KO lungs was diminished. This suggests that the function of CD44 as an adhesion molecule, regulating the migration and recruitment of leukocytes to sites of inflammation, but not its role as a signaling receptor and subsequent proinflammatory cytokine production is critical for the accumulation of inflammatory cells during CS aggravation of allergic inflammation (S4 Fig).

In conclusion, we demonstrate for the first time a crucial role for CD44 in a murine model of cigarette smoke-facilitated allergic airway inflammation.

## Supporting Information

S1 Fig Effect of CD44 on MHCII expression in dendritic cells upon combined HDM/CS exposure. Mean fluorescence intensity (MFI) of MHCII within the CD11c^+^, low autofluorescent, CD11b^+^ dendritic cells from in bronchoalveolar lavage fluid (BAL) of wild type (WT) and CD44 knockout (KO) mice that were exposed to either PBS/air, PBS/CS, HDM/air and HDM/CS (n: 8–10 mice/group, 8 groups).
(TIF)

S2 Fig Effect of CD44 on lymph node cytokine production upon combined HDM/CS exposure without HDM restimulation. Levels of IL-13, IFN-γ and IL-17 in supernatant of unstimulated lymph node cell cultures from WT and CD44 KO mice that were exposed for 3 weeks to PBS/air, PBS/CS, HDM/air or HDM/CS. (*p<0.05, **p<0.01, ***p<0.005; n = 8–10 mice/group, 8 groups).
(TIF)

S3 Fig Effect of CD44 on semi-quantitative pathology score upon combined HDM/CS exposure. Semi-quantitative pathology scoring on haematoxylin-eosin stained lung tissue from WT and CD44 KO mice that were exposed for 3 weeks to PBS/air, PBS/CS, HDM/air or HDM/CS (*p<0.05, **p<0.01, ***p<0.005; n: 8–10 mice/group, 8 groups).
(TIF)

S4 Fig Model of immunological mechanisms involving CD44 in the aggravation of allergic airway inflammation by cigarette smoke. The combination HDM/CS induces an inflammatory response with increased eosinophils, CD4^+^ T-cells, neutrophils, dendritic cells, Th2 cytokines, proinflammatory cytokines and goblet cells (increased cells and mediators are indicated in RED). In the absence of CD44, several inflammatory cells and Th2 cytokines are downregulated (indicated with black downward arrows), whereas proinflammatory mediators are not. Poor inflammatory cell recruitment in lungs of CD44 KO mice despite increased proinflammatory mediator release suggests a role of CD44 as adhesion molecule, rather than as signaling receptor in mediating CS-aggravated allergic airway inflammation to HDM.
(TIF)
