# Supplementary figures and images for: Aggravation of Allergic Airway Inflammation by Cigarette Smoke in Mice Is CD44-Dependent
Source: PLoS One. 2016 Mar 21;11(3):e0151113. doi: 10.1371/journal.pone.0151113 (PMC4801229; doi:10.1371/journal.pone.0151113)

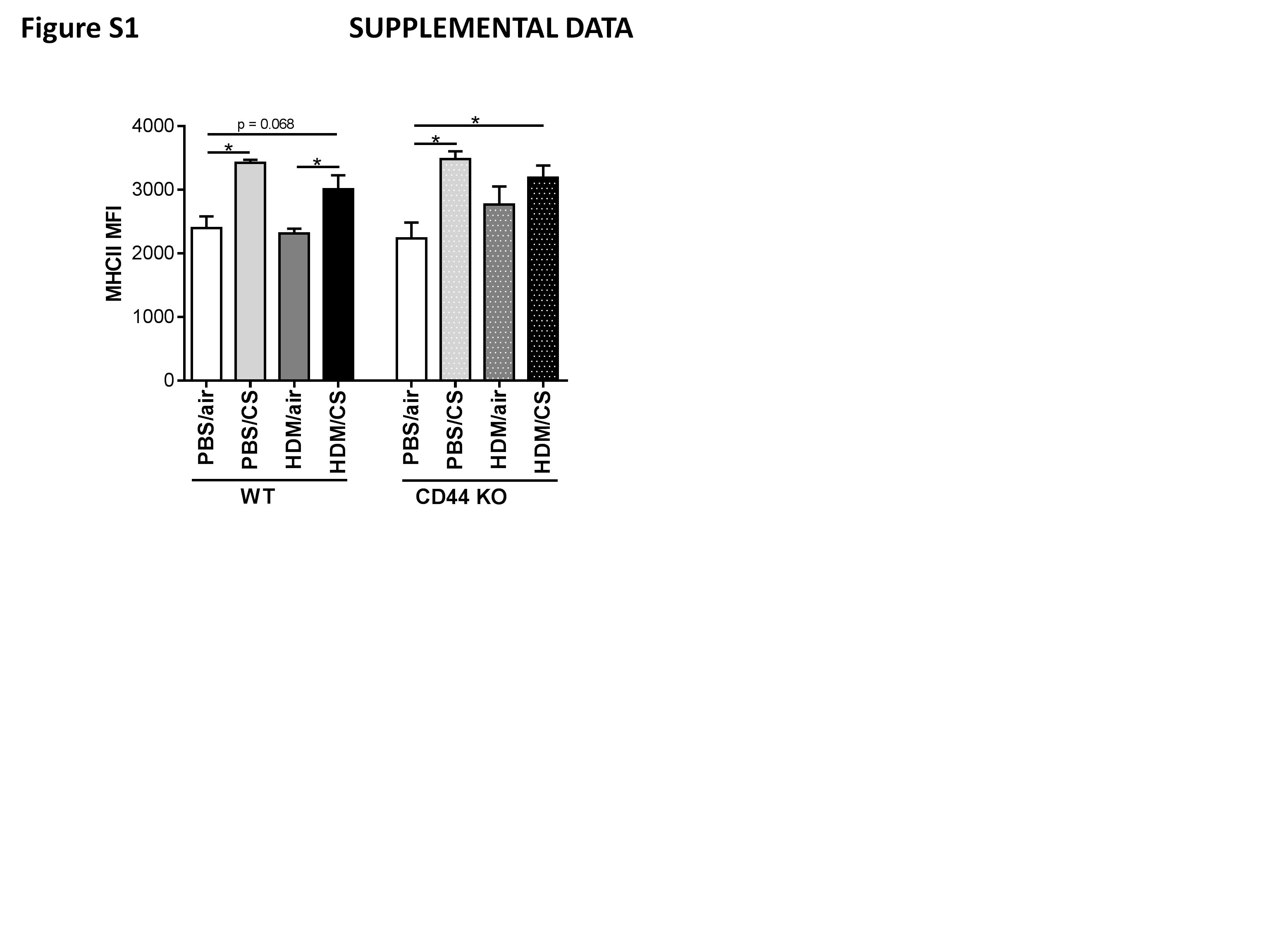

Supplement: S1 Fig — Mean fluorescence intensity (MFI) of MHCII within the CD11c+, low autofluorescent, CD11b+ dendritic cells from in bronchoalveolar lavage fluid (BAL) of wild type (WT) and CD44 knockout (KO) mice that were exposed to either PBS/air, PBS/CS, HDM/air and HDM/CS (n: 8–10 mice/group, 8 groups). (TIF) [file pone.0151113.s001.tif]

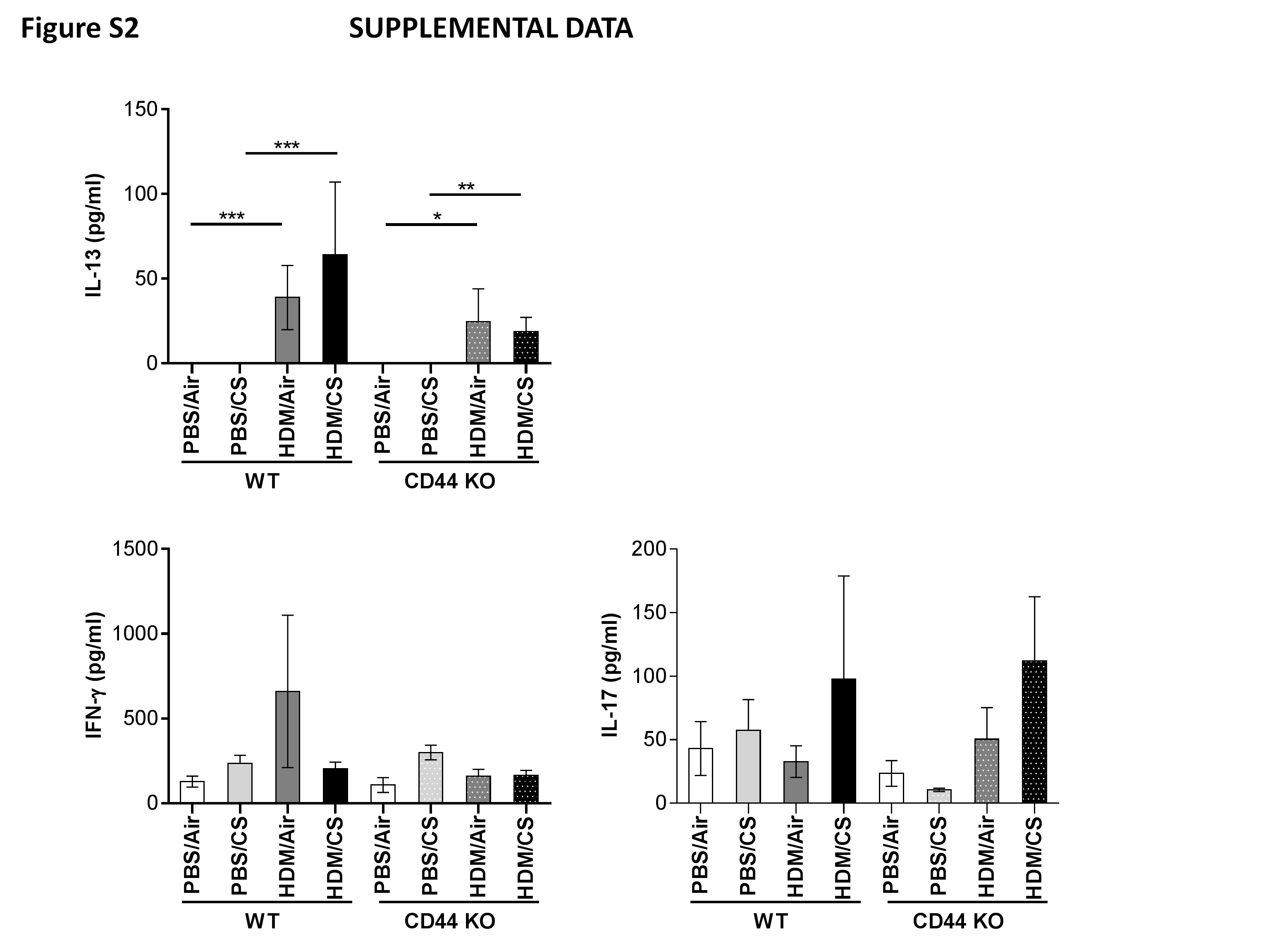

Supplement: S2 Fig — Levels of IL-13, IFN-γ and IL-17 in supernatant of unstimulated lymph node cell cultures from WT and CD44 KO mice that were exposed for 3 weeks to PBS/air, PBS/CS, HDM/air or HDM/CS. (*p<0.05, **p<0.01, ***p<0.005; n = 8–10 mice/group, 8 groups). (TIF) [file pone.0151113.s002.tif]

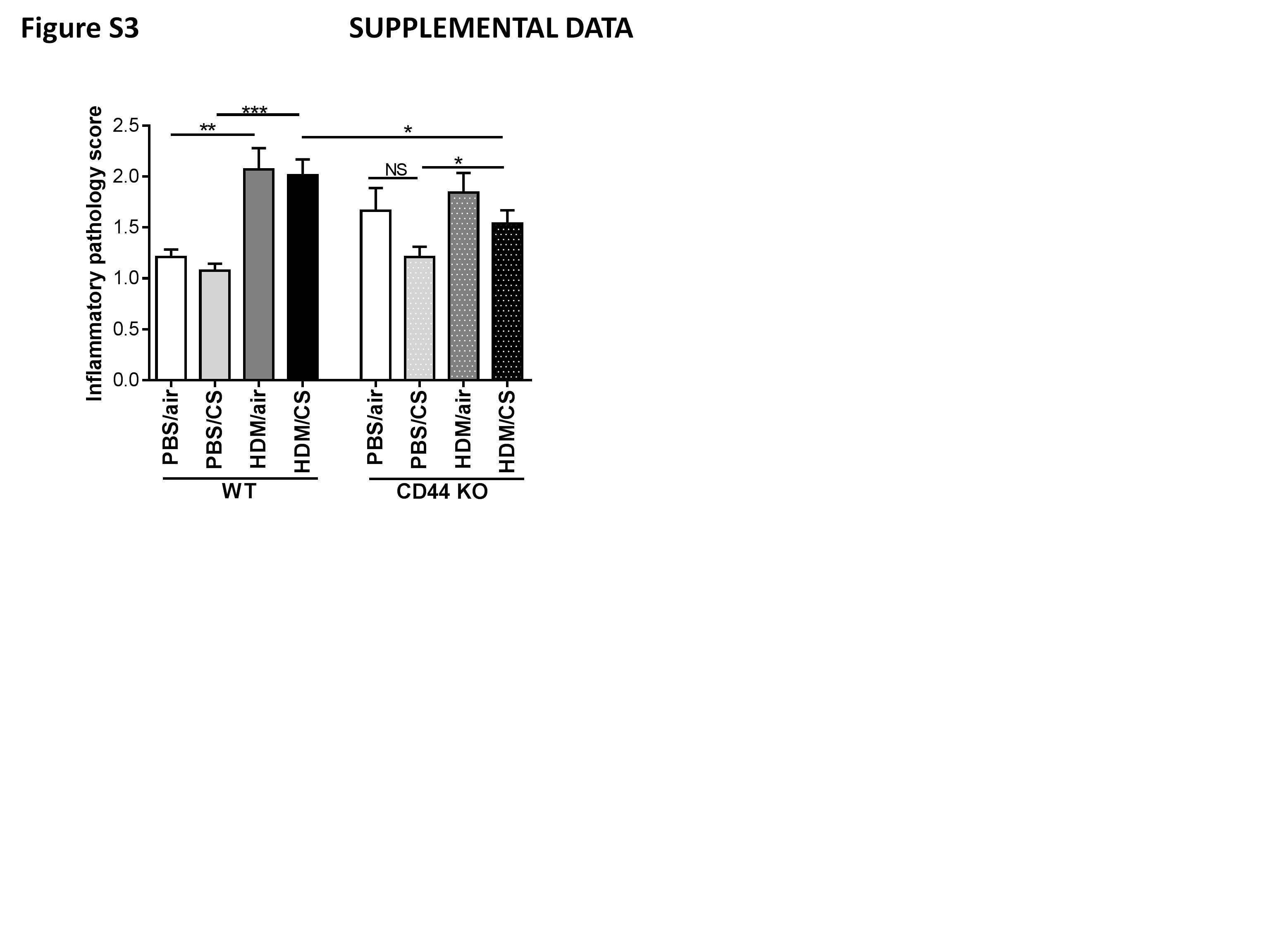

Supplement: S3 Fig — Semi-quantitative pathology scoring on haematoxylin-eosin stained lung tissue from WT and CD44 KO mice that were exposed for 3 weeks to PBS/air, PBS/CS, HDM/air or HDM/CS (*p<0.05, **p<0.01, ***p<0.005; n: 8–10 mice/group, 8 groups). (TIF) [file pone.0151113.s003.tif]

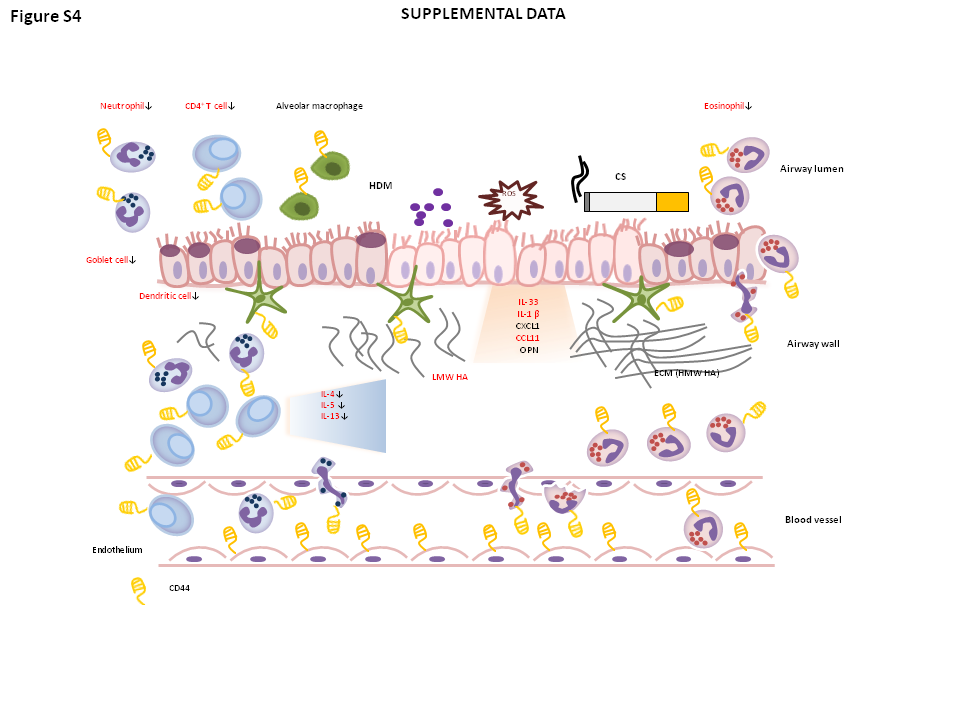

Supplement: S4 Fig — The combination HDM/CS induces an inflammatory response with increased eosinophils, CD4+ T-cells, neutrophils, dendritic cells, Th2 cytokines, proinflammatory cytokines and goblet cells (increased cells and mediators are indicated in RED). In the absence of CD44, several inflammatory cells and Th2 cytokines are downregulated (indicated with black downward arrows), whereas proinflammatory mediators are not. Poor inflammatory cell recruitment in lungs of CD44 KO mice despite increased proinflammatory mediator release suggests a role of CD44 as adhesion molecule, rather than as signaling receptor in mediating CS-aggravated allergic airway inflammation to HDM. (TIF) [file pone.0151113.s004.tif]
